# COVID-19 vaccination and miscarriage risk: RNA-seq and bioinformatics analysis at the maternal-foetal interface

**DOI:** 10.7189/jogh.15.04129

**Published:** 2025-11-14

**Authors:** Yiyuan Qu, Chengcheng Zhu, Tao Sun, Jianqiu Jiang, Ying Gu, Linping Jin, Xujia Huang, Bingbing Wu, Jian Xu, Xiuying Chen

**Affiliations:** 1 Department of Obstetrics-Gynecology, Center for Reproduction, the Fourth Affiliated Hospital, International Institutes of Medicine, Zhejiang University School of Medicine, Yiwu, Zhejiang, China; 2 Women's Hospital, Zhejiang University School of Medicine, Hangzhou, Zhejiang, China

## Abstract

**Background:**

Vaccine hesitancy persists because definitive evidence regarding the underexplored safety of COVID-19 vaccines in pregnancy is still lacking, particularly concerning their effects on the maternal-foetal interface (MFI) and potential links to miscarriage. We aimed to verify whether COVID-19 vaccines modulate gene expression at the MFI, thereby influencing recurrent miscarriage.

**Methods:**

We conducted an RNA sequencing analysis on decidual tissues from six pairs of early pregnancy participants, both vaccinated and unvaccinated. We extracted the data sets associated with COVID-19 placenta (GSE181238) and recurrent miscarriage (GSE22490) from the Gene Expression Omnibus database for further bioinformatic analysis, focussing on the expression, function, and distribution of core genes at the MFI.

**Results:**

Compared to the control group, 879 differentially expressed genes (*P* < 0.05; fold changes >1.5; false discovery rate <0.05) were identified in the vaccinated group. Complement activation and cell adhesion pathways were up-regulated, while the graft-vs-host response was down-regulated. The vaccine down-regulated some genes overexpressed in recurrent miscarriage cases. Three significant genes – FOS, FOSB, and LY96 – associated with miscarriage were identified; these genes are up-regulated during infection but suppressed by the vaccine. Functional enrichment analysis revealed the vaccine's immune activity, similar to but weaker than COVID-19 infection, and it inhibited certain miscarriage-related pathways, such as the tumour necrosis factor signalling pathway. Gene set variation analysis suggested a positive influence of the vaccine on immune tolerance at MFI.

**Conclusions:**

This study indicates that the COVID-19 vaccine may exert nonnegative effects on the maternal-foetal immune micro-environment and is unlikely to increase the risk of miscarriage.

COVID-19, caused by the SARS-CoV-2 virus, was initially found in Wuhan, China, in December 2019. Since then, it has spread to over 210 countries and territories worldwide, resulting in an ongoing global pandemic and causing extensive harm to human health [1]. Previous studies have shown that viral infection can lead to a variety of adverse outcomes during pregnancy, such as spontaneous abortion, premature birth, intrauterine foetal death and stillbirth [2]. Even multiple studies have confirmed that pregnant women infected with the novel coronavirus are at an increased risk of early miscarriage through inflammatory response, immune response, and complications [3]. Currently, there is no consensus on the impact of COVID-19 on the reproductive health of women.

COVID-19 vaccination is considered one of the best measures to prevent infection with the novel coronavirus. According to research from the World Health Organization and various national vaccine regulatory agencies, getting vaccinated against COVID-19 can obviously control the risk of infection and mitigate the symptoms and severity of COVID-19 [4]. Additionally, the COVID-19 vaccine provides a protective effect by activating the immune system and generating effective antibodies and memory cells to combat the invasion of the coronavirus [5]. Based on data from the World Health Organization, as of 10 March 2023, 13.8 billion COVID-19 vaccine doses have been administered globally [6]. Currently, as pregnant women are excluded from clinical drug trials of COVID-19 vaccines, there are only a few retrospective studies on the safety and effectiveness of the vaccines on the health of pregnant women and newborns, as well as mechanistic studies of vaccine responses in pregnant women [7]. Therefore, whether pregnant women should receive the COVID-19 vaccine and whether it leads to adverse pregnancy outcomes have raised global concerns. Several recent large-scale studies have reached similar conclusions that the risk of early pregnancy loss does not increase after COVID-19 vaccination [8]. These studies provide preliminary evidence supporting the safety of this vaccine for pregnant women. However, due to the complexity of biological reactions after vaccination and the process of pregnancy, the discussion on the specific mechanistic correlation between the COVID-19 vaccine and miscarriage is still lacking at present.

The maternal-foetal interface (MFI) means the contact surface between the mother and her fetus, including the decidua from the mother, the placenta from the fetus, the extravillous trophoblasts from the embryo, decidual stromal cells, and decidual immune cells [9]. The immunological balance is a critical factor in maintaining the function of the MFI. Moreover, the balance between immune activation and immunological tolerance towards embryonic antigens is essential for a successful pregnancy [10]. Immune dysregulation at the MFI can lead to miscarriage, preeclampsia, and other adverse pregnancy outcomes [11]. Some researchers showed that pregnant women contracting SARS-CoV-2 may encounter inflammation and immune dysregulation at the MFI. This can potentially lead to various adverse pregnancy outcomes like miscarriage, preterm birth, gestational hypertension, foetal growth restriction, and stillbirth [12]. It is believed that COVID-19 vaccination could help maintain immune balance at the MFI, reduce viral transmission, and decrease the risk of adverse pregnancy outcomes. Further confirmation and verification through basic experiments are still required to fully establish this conclusion.

In the current study, we established a clinical cohort to investigate the gene expression profile at the MFI after COVID-19 vaccine administration using next-generation RNA sequencing (RNA-seq). We also examined the gene expression data set related to COVID-19 infection and recurrent miscarriage through the Gene Expression Omnibus (GEO) database [13]. Subsequently, we conducted Gene set enrichment analysis (GSEA) using the vaccine RNA-seq matrix as a reference to identify differentially expressed genes (DEGs) related to COVID-19 or recurrent miscarriage and determined core enriched genes. Then, we performed functional analysis of the core enriched genes, including protein-protein interaction (PPI) construction and visualisation, Gene ontology (GO) and Kyoto Encyclopedia of Genes and Genomes (KEGG), and Gene set variation analysis (GSVA). Finally, we explored the expression and distribution of core-enriched genes at the MFI. We aimed to explore the potential relationship and specific biological mechanisms between COVID-19 vaccines and miscarriage at the MFI.

## METHODS

### RNA-seq

We selected 12 decidual tissue samples through random sampling from women who naturally conceived and voluntarily terminated their pregnancies between December 2021 and February 2022. Tissues were collected post-uterine curettage, cleaned, frozen in liquid nitrogen, and stored at −80°C. Participants were eligible for inclusion if they were pregnant women aged 20–40 with a singleton pregnancy, in the first trimester, and without known pregnancy complications. Participants were split into vaccinated (*i.e.* three doses post-last menstrual period) and unvaccinated control groups, with six members. We excluded participants with a history of smoking, pre-eclampsia, recurrent implantation failure, preterm birth, gestational diabetes, placental abnormalities, foetal anomalies, or chronic diseases (*e.g.* hypertension, diabetes, autoimmune disorders). Additionally, we excluded those who had used medications that could affect pregnancy outcomes. RNA was extracted, libraries prepared with Illumina's TruSeq kit, mRNA purified and fragmented, and cDNA concentration measured with Qubit 2.0 (Thermo). Library fragment lengths were determined with Agilent 2100, and RNA-seq was performed on the NovaSeq 6000. Bioinformatics analysis involved FastQC, version 0.11.9, for quality control, STAR aligner, version 2.7.10, for read alignment to hg38, and Cutadapt, version 4.3, for removing poor-quality reads. We performed quality control assessments at multiple stages, and the results indicated that our sequencing data were highly stable and reliable.

### Public dateset analysis

We used the GEO database, GeneCards database [14], Pubmed database [15], and MSigDB public database [16] to search, download, and analyse gene information related to COVID-19 infection, COVID-19 vaccines, miscarriage, and MFI. The data sets GSE181238 and GSE22490 were obtained from the GEO database of the National Center for Biotechnology Information. GSE22490 includes microarray data from placental tissues, samples were collected from women with recurrent miscarriage (*i.e.*≥3 consecutive pregnancy losses) and matched controls undergoing elective termination of uncomplicated pregnancies. In the GSE181238 data set, RNA-seq analysis was conducted on placental tissues from mothers who were COVID-19 positive (COVID-19+) and negative (COVID-1−). To ensure the reliability of the data, rigorous preprocessing and quality control measures were applied to these data sets. Samples underwent stringent filtering and normalisation procedures to eliminate technical biases. By analysing this information, we further identify the biological functions of core genes and cluster essential genes to gain a deeper understanding of their roles and interrelationships in specific biological processes or diseases.

### Bioinformatics analysis

We used DESeq2 package, version 1.38.3, from *R*, version 4.1 (R Core Team, Vienna, Austria) to explore differential gene expression. We excluded genes exhibiting low expression levels across all samples, with <10 counts. We defined statistical significance as a false discovery rate (FDR)<0.05 and fold changes (|FCs|)>1.5. We conducted functional analysis to evaluate the crucial genes' biological activities. We performed the GO and KEGG pathway analyses using the DAVID database [17], and the GO and KEGG analyses of differential genes (with screening criteria of FDR<0.1 and *P* < 0.05) using the clusterProfiler package, version 4.6.2, in *R*, along with the ggplot2 package, version 3.4.3, for visualisation. In addition, the PPI network is crucial for functional interactions between proteins, which helps identify central genes and potential biomarkers. We used the STRING [18], GeneMANIA [19], and Cytoscape [20] databases to construct and visualise the PPI network [21]. The STRING database provides essential computational predictions of protein interactions chains, GeneMANIA is used to predict gene functions and mappings, and Cytoscape provides data visualisation about molecular interaction networks and biological pathways, integrating these networks with annotations, gene expression profiles, and other state data. We employed the self-tested COVID-19 vaccine RNA-seq data set as the reference matrix for conducting GSEA analysis with the DEGs from GSE181238 and GSE22490. Furthermore, we conducted GSVA using the self-tested COVID-19 vaccine RNA-seq data as the reference and pathway-related immune infiltration, angiogenesis, and trophoblast cell migration sourced from the MSigDB database as targets. Ultimately, we investigated the expression and distribution of core-enriched genes at the MFI using the MFI data set [22], which we obtained from the PubMed database.

## RESULTS

The average age in our sample was 28 (standard deviation (SD) = 4.6), the average number of days since stopping menstruation was 55 (SD = 8.7), the average number of pregnancies was 3.6 (SD = 1.6), the average number of deliveries was 1 (SD = 0.8), the average body mass index was 19.6 (SD = 1.9), and all participants were of Asian ethnicity (Table S1 in the **Online Supplementary Document**).

### COVID-19 vaccination and MFI

**Figure 1:**
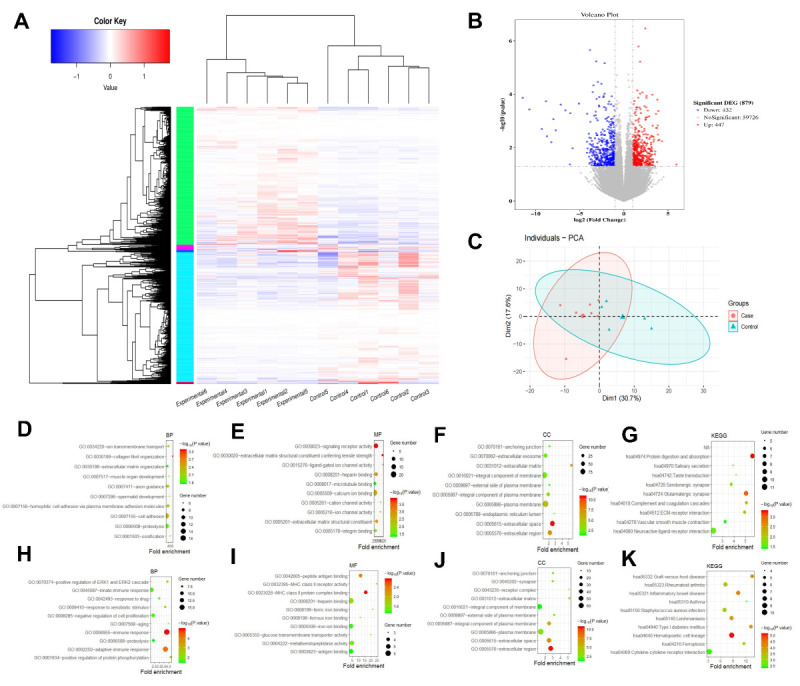
Differential gene expression identification and functional enrichment analysis between the vaccinated and unvaccinated groups. **Panel A.** Heatmap of DEGs. **Panel B**. Volcano plot of DEGs. **Panel C.** Principal component analysis plot of DEGs. **Panel D.** Bubble chart of the biological processes of the up-regulated genes. **Panel E.** Bubble chart of the molecular function of the up-regulated genes. **Panel F.** Bubble chart of the cellular component of the up-regulated genes. **Panel G.** Bubble chart of KEGG of the up-regulated genes. **Panel H.** Bubble chart of the biological processes of the down-regulated genes. **Panel I.** Bubble chart of the molecular function of the down-regulated genes. **Panel J.** Bubble chart of the cellular component of the down-regulated genes. **Panel K.** Bubble chart of KEGG of the down-regulated genes. DEG – differentially expressed gene.

We used the decidual tissues of six early pregnancy patients who had received three complete doses of the COVID-19 vaccine as the experimental group and the decidual samples from six patients of the same period who had not received the COVID-19 vaccine as the control group. RNA-seq results showed meaningful differences in gene expression between the two groups. A total of 879 DEGs were identified, with 447 genes up-regulated and 432 genes down-regulated (*P* < 0.05; FDR<0.05) (**Figure 1****,** Panels A–C). Focussed on the top 500 significant transcripts with adjusted *P*-values, we analysed their biological features by conducting GO and KEGG analyses (Tables S2 and S5 in the **Online Supplementary Document**).

The visualised analysis revealed the top ten GO and KEGG terms (**Figure 1****,** Panels D–L). For the up-regulated group of genes, the GO analysis showed that complement activation and cell adhesion were the most enriched biological processes. Regarding cellular component, DEGs were mainly enriched in the plasma membrane and anchoring junction. Regarding molecular function, the results indicated that signalling receptor activity was significantly enriched in the up-regulated genes. In the KEGG pathway analysis, the up-regulated DEGs were notably enriched in complement cascades. On the other hand, the down-regulated genes were significantly enriched in immune response-related biological processes, including innate immune response and adaptive immune response. The cellular component analysis revealed that the down-regulated DEGs were mainly enriched in the plasma membrane. Regarding molecular function, the down-regulated DEGs were notably enriched in antigen binding and MHC class II protein complex binding. The KEGG pathway analysis showed that the down-regulated DEGs were predominantly enriched in cytokine-cytokine receptor interaction, graft-vs-host disease, rheumatoid arthritis, and inflammatory bowel disease, which are highly related to immune response (Table S3 in the **Online Supplementary Document**).

### COVID-19 infection and MFI

**Figure 2:**
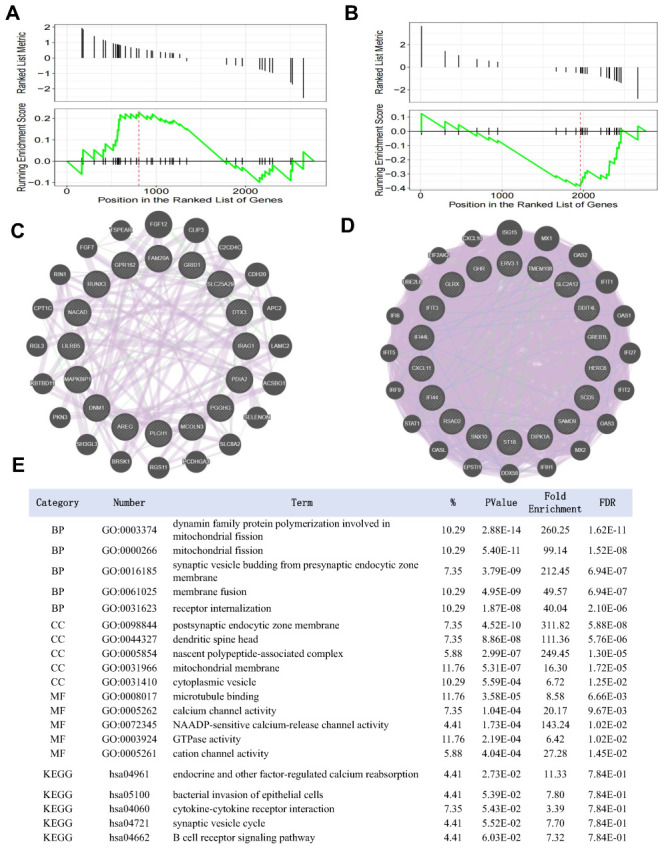
Systematical analysis of the COVID-19 infection and MFI based on GSE181238. **Panel A.** The GSEA plot reveals that the RNA-seq matrix exhibited a concentration of the up-regulated placenta gene set in relation to COVID-19 infection. **Panel B.** The GSEA plot reveals that the RNA-seq matrix exhibited a concentration of the down-regulated placenta gene set in relation to COVID-19 infection. **Panel C.** The PPI network of the up-regulated core-enrichment genes relation to COVID-19 infection in the GeneMANIA database. **Panel D.** The PPI network of the down-regulated core-enrichment genes relation to COVID-19 infection in the GeneMANIA database. **Panel E.** The functional enrichment analysis of core-enrichment placenta genes relation to COVID-19 infection. MFI – maternal-foetal interface.

The data set GSE181238 included transcriptome data from placenta samples collected at delivery time. It consisted of five samples from healthy controls, four from individuals with COVID-19, and four from individuals with other inflammatory pathologies. The gene expression profiles were obtained using the Illumina NovaSeq 6000 platform (Homo sapiens) with the GPL24676 annotation. We downloaded and curated the expression data set of GSE181238 and identified 24 up-regulated genes and 56 down-regulated genes associated with COVID-19 infection (Table S6 in the **Online Supplementary Document**). To further investigate the potential association between SARS-CoV-2 infection and the impact of COVID-19 vaccines at the MFI, we used RNA-seq expression matrices after vaccination as reference data and performed a GSEA analysis using 24 up-regulated and 56 down-regulated genes associated with viral infections. The GSEA results showed that most up-regulated COVID-19 vaccine genes are mainly enriched at the top of the GSE181238 gene list. Furthermore, many down-regulated genes were enriched in the bottom half of the list (**Figure 2**, Panels A and B). In the GSEA analysis of COVID-19 vaccines, we identified 16 up-regulated core enrichment genes. These genes include PDIA2, GRID1, AREG, IRAG1, NACAD, MCOLN3, FAM20A, MAPK8IP1, DNM1, PLCH1, SLC25A29, RUNX3, PGGHG, GPR162, DTX3, and LILRB5. Similarly, 19 core enrichment genes were identified as down-regulated. These genes are SNX10, SCD5, IFIT3, GHR, HERC6, IFI44, SAMD9, DIPK1A, GLRX, RSAD2, TMEM108, PKN2-AS1, ERV3-1, DDIT4L, GREB1L, ST18, CXCL11, IFI44L, and SLC2A12 (Table S4 in the **Online Supplementary Document**). Using the GeneMANIA platform, we constructed a hypothetical central PPI network. In addition to the 16 up-regulated core enrichment genes and 19 down-regulated core enrichment genes, were 40 genes closely related to the core enrichment genes in terms of co-expression, pathway interactions, physical interactions, and predictions (**Figure 2**, Panels C and D). Finally, we conducted an in-depth investigation of the functions of the key genes through GO and KEGG pathway enrichment analysis. The results indicated significant enrichment in receptor internalisation, nascent polypeptide-associated complex, GTPase activity, and cytokine-cytokine receptor interaction (**Figure 2**, Panel E).

### Recurrent miscarriage and the MFI

**Figure 3:**
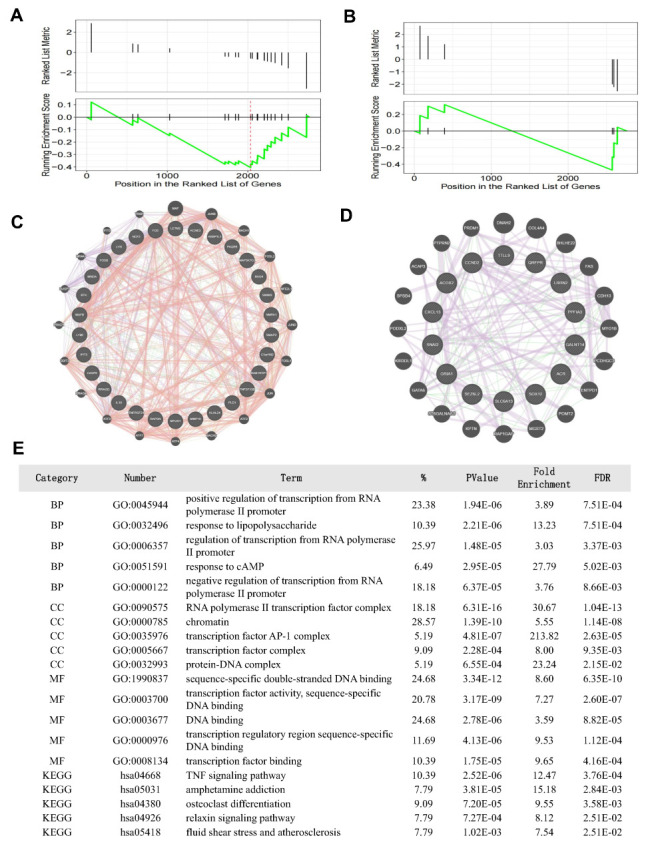
Systematical analysis of the recurrent miscarriage and MFI based on GSE22490. **Panel A.** The GSEA plot reveals that the RNA-seq matrix exhibited a concentration of the up-regulated placenta gene set in relation to recurrent miscarriage. **Panel B.** The GSEA plot reveals that the RNA-seq matrix exhibited a concentration of the down-regulated placenta gene set in relation to recurrent miscarriage. **Panel C.** The PPI network of the up-regulated core-enrichment genes relation to recurrent miscarriage in the GeneMANIA database. **Panel D.** The PPI network of the down-regulated core-enrichment genes relation to recurrent miscarriage in the GeneMANIA database. **Panel E.** The functional enrichment analysis of core-enrichment placenta genes relation to recurrent miscarriage. MFI – maternal-foetal interface.

The GSE22490 data set, based on the Affymetrix GeneChips platform (GPL570), profiles placental tissue samples from six elective terminations and four recurrent miscarriages. It identified 131 up-regulated and 108 down-regulated genes linked to recurrent miscarriage (Table S7 in the **Online Supplementary Document**). Using COVID-19 vaccine RNA-seq data as a reference, GSEA analysis was conducted with these genes, revealing that most up-regulated COVID-19 vaccine genes were enriched at the bottom of the GSE22490 list, while down-regulated genes were in the top half (**Figure 3**, Panels A and B).

This analysis identified 32 up-regulated core enrichment genes and 16 down-regulated ones. The up-regulated genes include KLHL24, KCNE3, C1orf162, MAP3K7CL, HSBP1L1, and others, while the down-regulated genes comprise QRFPR, ACR, CXCL13, SLC6A13, GRIA1, and more (Table S7 in the **Online Supplementary Document**). Using GeneMANIA, a PPI network was constructed for these core genes and their closely related ones, co-expressed and interacting within pathways (**Figure**, Panels C and D). The most significant biological process was positive regulation of transcription, the essential component was the transcription factor complex, and the indispensable molecular function was transcription factor activity. The tumour necrosis factor (TNF) signalling pathway was identified as the most important KEGG pathway (**Figure 3**, Panel E).

### Gene set enrichment analysis

To investigate the impact of COVID-19 vaccination on gene expression at the MFI, we used gene sets from MSigDB related to immune infiltration, angiogenesis, and trophoblast cell migration. GSVA analysis with COVID-19 vaccine RNA-seq data as the reference identified 1854 immune infiltration gene sets, with 200 associated with vaccination and 72 significant, including CD40 and CXCR4. For angiogenesis, 66 gene sets were related to vaccination, though none were significant. Trophoblast cell migration showed 16 significant gene sets related to vaccination, with chorionic trophoblast cell proliferation being significant.

We also analysed the expression of 32 up-regulated and 16 down-regulated core genes linked to recurrent miscarriage at the MFI and found varied expression at the cellular level. Similarly, 16 up-regulated and 19 down-regulated core genes related to COVID-19 mainly showed low expression, except for some highly expressed genes like DC1 and dNKs.

Further research on 17 core enriched genes expressed at the MFI indicated that CXCL11, MMP10, FOS, FOSB, LY96, and NCF2 may be closely related to miscarriage. Notably, COVID-19 vaccination down-regulated the expression of these genes compared to the non-vaccinated control, with FOS, FOSB, and LY96 showing significant differences (Figure S1 in the **Online Supplementary Document**).

## DISCUSSION

Multiple studies have shown that pregnant women infected with the SARS-CoV-2 are at an increased risk of adverse outcomes [23]. The inflammatory and immune responses triggered at MFI by the virus can potentially lead to various complications, including early miscarriage [24]. COVID-19 vaccination is considered one of the best preventive measures against SARS-CoV-2 infection. Existing studies indicate that there is no evidence supporting an increased risk of early miscarriage after COVID-19 vaccination [25]. A retrospective study suggests that receiving the COVID-19 mRNA vaccine during pregnancy may reduce stillbirth cases [26]. However, as a new approach, it may still cause specific side effects in individuals, including pregnant women [27]. Although some studies support that vaccination is relatively safe concerning delivery complications, the series of immune responses triggered by the COVID-19 vaccine may lead to unknown adverse effects on the foetus by affecting the placental basal decidua [28]. In this study, we used RNA-Seq and bioinformatics techniques to explore the potential correlation and specific biological mechanisms linking COVID-19 vaccines to the risk of miscarriage at the MFI.

To date, several studies have investigated the impact of the COVID-19 virus on the placenta and MFI. Current histopathological analyses have identified the presence of viral particles, inflammation, and poor vascular perfusion in the placentas of COVID-19 patients [29]. Other studies have explored the cellular targets for viral entry into the placenta and the altered molecular signals [30]. However, despite the pivotal role of decidual tissue in forming the placenta, there remains a lack of research focusing on the link between vaccination and decidual changes during the early stages of pregnancy.

In our study, we discovered a total of 879 DEGs following COVID-19 vaccination. Among these DEGS, 447 genes were up-regulated while others were down-regulated. These DEGs enriched several biological pathways, including protein digestion and absorption, glutamatergic synapse, complement and coagulation cascades, cytokine-cytokine receptor interaction, graft-vs-host disease, and rheumatoid arthritis. Furthermore, most up-regulated genes associated with recurrent miscarriages are predominantly enriched in the lower part of the vaccine RNA-seq data set. This suggests that the COVID-19 vaccine has a similar but weaker immunogenicity than a virus infection and has no apparent negative impact on miscarriages.

This study further investigated and explored 83 core enriched genes identified by GSEA through in-depth screening and analysis of public databases and single cell sequencing data. The study found that the most relevant signalling pathways associated with miscarriage include the TNF signalling pathway and mitogen-activated protein kinases (MAPK) signalling pathway. The associated genes include CXCL11, MMP10, FOS, FOSB, LY96, and NCF2. The MAPK signalling pathway can regulate the functions of trophoblast cells, such as proliferation, differentiation, and invasion. Dysfunction in this pathway may contribute to recurrent spontaneous abortion [31]. Another highly enriched pathway, the TNF signalling pathway, contributes to immune regulation and inflammation. Excessive levels of TNF-alpha have been implicated in complications during pregnancy, such as recurrent spontaneous abortion and recurrent implantation failure [32]. Previous studies have shown that CXCL11 is essential in immune regulation at the MFI by modulating the balance of Th1 and Th2 cells or inhibiting excessive invasion of the trophoblast layer [33]. MMP10 was involved in remodelling placental tissue and degradation of the extracellular matrix. It also regulates pathways such as cell apoptosis, inflammatory response, and angiogenesis, which are closely associated with miscarriage [34]. FOS was known to be involved in both the MAPK and TNF signalling pathways. FOS contributes to promoting the proliferation of extravillous trophoblast cells and controlling trophoblast invasion. FOS also plays a role in adjusting the MMP1, MMP3, and MMP10 expression, which are involved in remodelling the extracellular matrix and can impact placenta implantation and formation [35]. Similarly, FOSB has a similar role to FOS in these processes [36]. Also known as myeloid differentiation two, LY96 is necessary for the toll-like receptor four activation by lipopolysaccharides, playing an important role in the innate immune response. It is associated with the pro-inflammatory cytokines and type I interferons' production, and it is also associated with adverse pregnancy outcomes [37]. One past study used transcriptome analyses to compare gene expression differences in the maternal part of the placenta between women with infection symptoms and asymptomatic women, revealing alterations in pathways associated with oxidative phosphorylation. These molecular findings align with our research results. We observed a down-regulation in NCF expression following vaccination [38]. NCF2 maintains oxidative-antioxidative balance and immune tolerance through mediating oxidative phosphorylation processes. Overexpression of NCF2 can lead to excessive reactive oxygen species production, promote the expression of metalloproteinases, disrupt the balance between metalloproteinases and tissue inhibitors, and impair oxidative stress or antioxidant production [39]. This study confirms through GSVA that the COVID-19 vaccine has a notable influence on immune tolerance at the MFI and shows that it does not significantly affect angiogenesis and trophoblast cell migration. We found that important genes associated with miscarriage exhibit significantly reduced expression following COVID-19 vaccination. We identified and confirmed the genetic changes induced by the vaccine at the MFI in miscarriage cases; however, the impact of these genetic alterations on prior immune responses remains to be elucidated.

Compared to previous studies, our study focussed on the decidua in the early stage of pregnancy as the research subject. This allows us to explore the vaccine's impact on the uterine microenvironment at MFI in the early stage. Furthermore, we further researched the link between recurrent miscarriages and COVID-19 vaccination. Our research innovatively indicates that the COVID-19 vaccine may not increase the risk of miscarriage at the MFI and may have a nonnegative effect.

However, validation of the interactions between various genes and previous immunity is still needed. Due to the relatively small sample size, our findings apply only to a subset of Asian women of childbearing age. Further research is needed to include women of different races and age groups. While we have presented meaningful insights into COVID-19 vaccines, future research will still require expanding sample sizes to validate the molecular biomarkers we have discovered and relevant in vitro experiments. Furthermore, clinical and basic research is needed to explore the specific mechanisms involved.

## CONCLUSIONS

Although the COVID-19 vaccine can exert several changes on the immune microenvironment at the MFI, our preliminary evidence does not support an increased risk of miscarriage associated with the COVID-19 vaccination.

## Additional Material


Online Supplementary Document
