# Supplementary figures and images for: COVID-19 vaccination and miscarriage risk: RNA-seq and bioinformatics analysis at the maternal-foetal interface
Source: J Glob Health. 2025 Nov 14;15:04129. doi: 10.7189/jogh.15.04129 (PMC12615006; doi:10.7189/jogh.15.04129)

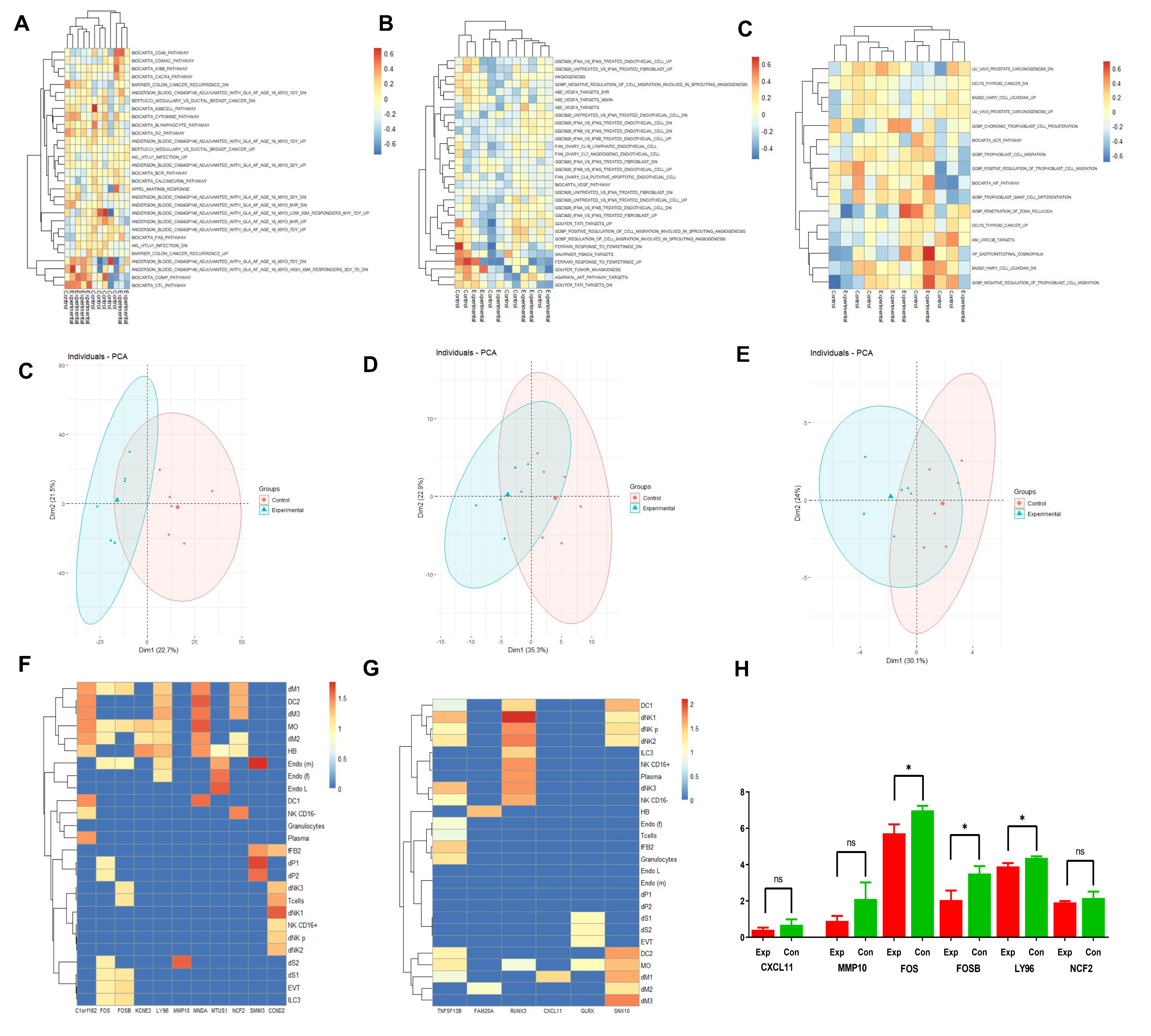

Supplement: Online Supplementary Document [file jogh-15-04129-s001.zip › jogh-15-04129-s001.tiff]
